# External Evaluation of Population Pharmacokinetic Models of Methotrexate for Model-Informed Precision Dosing in Pediatric Patients with Acute Lymphoid Leukemia

**DOI:** 10.3390/pharmaceutics15020569

**Published:** 2023-02-08

**Authors:** Shengfeng Wang, Qiufen Yin, Minghua Yang, Zeneng Cheng, Feifan Xie

**Affiliations:** 1 Division of Biopharmaceutics and Pharmacokinetics, Xiangya School of Pharmaceutical Sciences, Central South University, Changsha 410013, China; 2 Postdoctoral Research Station of Clinical Medicine and Department of Pediatrics, The Third Xiangya Hospital, Central South University, Changsha 410013, China; 3 Department of Pharmacy, The Third Xiangya Hospital, Central South University, Changsha 410013, China

**Keywords:** methotrexate, children, acute lymphoid leukemia, population pharmacokinetics, external validation

## Abstract

Background: Methotrexate (MTX) is a key immunosuppressant for children with acute lymphoid leukemia (ALL), and it has a narrow therapeutic window and relatively high pharmacokinetic variability. Several population pharmacokinetic (PopPK) models of MTX in ALL children have been reported, but the validity of these models for model-informed precision dosing in clinical practice is unclear. This study set out to evaluate the predictive performance of published pediatric PopPK models of MTX using an independent patient cohort. Methods: A PubMed literature search was performed to identify suitable models for evaluation. Demographics and measurements of the validation dataset were retrospectively collected from the medical records of ALL children who had received intravenous MTX. Predictive performance for each model was assessed by visual comparison of predictions to observations, median and mean predicted error (PE), and relative root mean squared error (RMSE). Results: Six models were identified for external evaluation, carried out on a dataset containing 354 concentrations from 51 pediatrics. Model performance varied considerably from one model to another. Different models had the median PE for population and individual predictions at −33.23% to 442.04% and −25.20% to 6.52%, mean PE for population and individual predictions at −25.51% to 780.87% and 1.33% to 64.44%, and RMSE for population and individual predictions at 62.88% to 1182.24% and 63.39% to 152.25%. All models showed relatively high RMSE. Conclusions: Some of the published models showed reasonably low levels of bias but had some problems with imprecision, and extensive evaluation is needed before model application in clinical practice.

## 1. Introduction

Methotrexate (MTX), a folate reductase inhibitor, has been widely used in the treatment of autoimmune diseases and malignancies for decades [1,2]. Leukemia is a group of malignant neoplastic diseases of the hematopoietic system, and it poses a significant health risk to children. Childhood leukemia mainly includes acute lymphoid leukemia (ALL) and acute myeloid leukemia (AML). ALL is the most common childhood cancer [3], and it accounts for more than 70% of childhood leukemia in China [4]. MTX is the cornerstone of maintenance cycles in childhood ALL chemotherapy [5], and its clinical dose can be roughly classified as low (<0.5 g/m^2^), intermediate (0.5–1.0 g/m^2^), and high (>1.0 g/m^2^) dose [6]. High-dose MTX (HD-MTX) provides the ability to target extramedullary leukemia by producing cytotoxic concentrations in sanctuary sites (e.g., cerebrospinal fluid) where low-dose MTX does not readily distribute [7]. Compared with low-dose MTX, HD-MTX shows significantly better patient event-free survival for childhood leukemia [8,9], and the use of HD-MTX is widely accepted as the first-line therapy in consolidation for childhood ALL [10,11].

In clinical practice, administration of HD-MTX often begins with a loading dose (e.g., 10% of the total dose) infused over 0.5 h, followed immediately by a continuous titration of the remaining dose over 23.5 h. It has been described that the steady-state plasma MTX concentration (C_p,ss,_ the concentration after the end of 24 h MTX infusion) is most appropriately associated with its therapeutic efficacy [12]. Depending on the risk level of cancer patients, the dose level of HD-MTX varies individually for maximizing efficacy while minimizing the toxicities [13]. For instance, a relatively high dose (e.g., 5.0 g/m^2^) is suggested for ALL children with intermediate risk (IR) or high risk (HR) in order to improve the outcome [14], while the relatively low dose (e.g., 3.0 g/m^2^) is adequate for low risk (LR) ALL children [10]. Age, leukocyte count, leukemic cell genotype, and minimal residual disease (MRD) are the important factors for risk classification [14,15]. The MTX dose is deemed effective if the C_p,ss,_ is above 65 μmol/L (IR and HR patients) or 33 μmol/L (LR patients) [11,12], and the treatment is subtherapeutic with a high risk of relapse when the C_p,ss,_ is below < 16 μmol/L. HD-MTX is associated with a variety of toxic reactions, and liver injury and nephrotoxicity are two of the serious adverse events [5,16]. It has been reported that MTX concentrations > 100 μmol/L at 24 h, >1.0 μmol/L at 48 h, and >0.1 μmol/L at 72 h are supratherapeutic and are associated with increased toxicity [10,17,18]. Therefore, the optimal use of HD-MTX is an important question for providing balanced efficacy and toxicity.

MTX has a narrow therapeutic window, and the pharmacokinetics (PK) of HD-MTX shows wide inter-individual variability (IIV) in children even under the same dosing regimen [19,20]. Routine therapeutic drug monitoring (TDM) for HD-MTX has been strongly recommended to reduce the incidence of adverse events in the target patient population [19,21]. Population pharmacokinetic (PopPK) models have the potential to improve patient care by streamlining the TDM process [22]. PopPK models are less dependent on precisely timed drug concentrations than traditional peak and/or trough concentration-based monitoring, and they provide the options to quantify PK variability and identify clinical characteristics (e.g., age and renal function) affecting the drug’s PK. In particular, for the immunosuppressive agents that are typically personalized by TDM, PopPK models allow for the area under the concentration-time curve (AUC) guided monitoring using Bayesian forecasting with limited sampling. Furthermore, PopPK models can aid in accelerating the initial dose titration through a priori model-informed precision dosing (MIPD) based on the baseline covariates of the target patients. Further optimization of subsequent treatment target attainment can be performed through a posteriori MIPD based on available TDM measurements and updated covariates over time [23,24].

MIPD is an advanced quantitative approach (e.g., PopPK model) that combines prior knowledge on the drug-disease-patient system with patient data from TDM to support individualized dosing in ongoing treatment [25], which takes into account inter-individual variability in a drug’s exposure. By combining the efficacy and toxicity risk targets, MIPD offers the potential to improve initial dose selection and individualized dose optimization, thereby it can minimize the duration of subtherapeutic or supratherapeutic HD-MTX doses. Before considering a PopPK model used in MIPD, model evaluation is an essential step to assess the model’s accuracy and predictive performance. The most crucial question in the evaluation of a PopPK model is if it can extrapolate the model’s results to patients at other institutions or forecast the drug’s PK in prospective studies, especially when the goal of the model is to determine the ideal individual dose. Internal evaluation is necessary to test a model’s ability to describe the population with which it has been constructed, and an external evaluation must be carried out to assess the predictive performance of the model when extrapolated. Currently, the PopPK models of HD-MTX in ALL children have been extensively reported [11,18,19,20,21,26], while the reported PK parameters and identified covariates varied significantly. Although some of the published PopPK models of MTX in ALL children have been internally evaluated with a dataset from the same patient cohort, this does not guarantee a good prediction performance when subjected to similar patient populations from external institutions [27]. Therefore, it is important to evaluate the predictive performance of the published PopPK models of MTX using independent datasets in order to identify the best candidate model for MIPD.

The aim of this study was to provide a systemic summary of the published PopPK models of MTX in ALL children and evaluate the predictive performance of these models using an independent dataset.

## 2. Methods

### 2.1. Literature Search for the PopPK Models of MTX in ALL Children

A search of the literature data for the PopPK models of MTX in ALL children was made through PubMed (until 30 November 2022) using the following search terms: (population pharmacokinetics [Title/Abstract]) AND (methotrexate [Title/Abstract]) AND (children [Title/Abstract] OR paediatrics [Title/Abstract]). The identified studies were reviewed, and the relevant references were examined to identify other potential publications for inclusion. Published studies were included for further analysis if the PopPK of MTX was conducted in children with ALL. PopPK models of MTX were excluded from the external validation if (1) the key information (e.g., the typical PK parameters and IIVs) is inadequate for the model recompilation; and (2) the mean or median age of the patient population is beyond the age range (1.0–13.0 years) of our patient cohort for the external validation. For eligible PopPK models, the following information was extracted from the original studies: structure of the compartmental model, population PK estimates, covariate model, inter- and intra-individual variability, residual variability, and estimation method.

### 2.2. Dataset for External Evaluation

Data were collected retrospectively from the medical records of patients treated with HD-MTX at the Third Xiangya Hospital at Central South University from 2019 to 2022. This study was approved by the ethics committees of the hospital and was registered at the Chinese Clinical Trial Registry (Register number: ChiCTR2000035264). Throughout the study, all the procedures were carried out in accordance with the Declaration of Helsinki. ALL was diagnosed if at least 25% of lymphoblasts were present in the bone marrow [15], and we included patients with ALL who were under 18 years of age at the time of diagnosis.

Children with ALL were classified as LR, IR, and HR. The children with ALL whose MRD was <1% at 19 days or MRD < 0.01% at 46 days were placed in the LR group. Patients whose MRD was ≥1% at 46 days or who had leukemia blasts ≥ 5% without MRD markers or with mixed lineage leukemia fusion genes, age < 6 months, and WBC ≥ 300 × 10^9^/L were placed in the HR group. All other cases are placed in the IR group. Theoretically, patients in the LR group were given an HD-MTX dose of 3.0 g/m^2^, and patients in the IR or HR group were given an HD-MTX dose of 5.0 g/m^2^. In practice, the actual HD-MTX dose was decided by the physicians for each patient. MTX was intravenously administered within 0.5 h for 10% of the total dose, and the remaining 90% dose was infused over 23.5 h.

Plasma samples were collected every 24 h until the MTX concentration dropped below 0.1 μmol/L. The actual sampling times were determined by the responsible nurses and may be slightly deviated from the theoretical points. MTX concentration was determined using fluorescence polarization immunoassay with a quantification limit of 0.05 μmol/L. Data were included if at least one intravenous dose of MTX and the concentration measurements were available. Data were excluded if there was any uncertainty about the time of dosing, infusion duration, or the sampling time of concentration measurements. Baseline characteristics of the ALL children were collected, including the demographics (gender, age, weight, body surface area (BSA), and height), the laboratory measurements (white blood cells, neutrophils, serum creatinine (Scr), alanine aminotransferase (ALT), and aspartate aminotransferase (AST)), and the estimated glomerular filtration rate (eGFR) based on the Bedside Schwartz formula (Equation (1)) [28]. Data collation was performed using R version 4.2.0 (R Foundation for Statistical Computing, Vienna, Austria). No patient data in the external validation dataset had previously been included in the development of any of the PopPK models of MTX.

(1) $$\begin{array}{c} \mathrm{eGFR}=\frac{0.413\times \mathrm{height}}{\mathrm{Scr}} \end{array}$$

### 2.3. Evaluation of Predictive Performance

Each selected PopPK model was separately implemented in NONMEM^®^ software (version 7.3, Icon Development Solutions, Ellicott City, MD, USA) as described in the original article. Parameter estimates and covariate models for each PopPK model were set to those determined in the original publications. Concentration predictions were generated based on the specific PopPK models in combination with the doses, sampling times, and covariates recorded in the validation dataset. That is, the predicted concentrations were obtained based on the PopPK models using the maximum a posteriori-Bayesian estimation (MAP-BE, $ESITIMATION MAXEVAL=0 in NONMEM). Reported PK parameters were adopted for each PopPK model to calculate the predicted population and individual concentrations at sampling times identical to those of our own data. If the specified covariate in the PopPK model was not available in the evaluation dataset, the mean or median covariate value was used as stated in the original publication. In addition, a proportional error of 30% was assumed for the residual variability if the models did not report this information.

The predictive performance of the PopPK models was assessed by the use of graphics and statistical metrics. Goodness-of-fit (GOF) was assessed graphically by comparing observed concentrations (C_obs_) to population predicted concentrations (C_pred_) and individual predicted concentrations (C_ipred_) to inspect potential bias (a systematic upward or downward deviation from the line of unity) and imprecision (a high degree of scattered data points around the line of unity). Furthermore, the Bland-Altman (B-A) plots were created to visualize the trends in bias for the population and individual predictions. The prediction error (PE; in percent; Equation (2)), the mean relative error (MPE; in percent; Equation (3)), and the relative root mean squared error (RMSE; in percent; Equation (4)) were calculated for population and individual predictions to quantify the predictive performance.

(2) $$\begin{array}{c} \mathrm{PE}=\frac{C_{i,\mathrm{pred}}-C_{\mathrm{obs}}}{C_{\mathrm{obs}}} \end{array}$$

(3) $$\begin{array}{c} \mathrm{MPE}=\frac{1}{N}\overset{N}{\underset{i=1}{\sum }}\frac{C_{i,\mathrm{pred}}-C_{\mathrm{obs}}}{C_{\mathrm{obs}}} \end{array}$$

(4) $$\begin{array}{c} \mathrm{RMSE}=\sqrt{\frac{1}{N}\overset{N}{\underset{i=1}{\sum }}{\left(\frac{C_{i,\mathrm{pred}}-C_{\mathrm{obs}}}{C_{\mathrm{obs}}}\right)}^{2}} \end{array}$$

where N is the number of observed MTX concentrations, and C_i,pred_ is the C_ipred_ or C_pred_ of MTX.

A PopPK model was deemed acceptable for our clinical settings when both the mean and median values of PE were less than 20% [27,29] and RMSE was less than or equal to 30% [29,30]. Data processing and plotting were carried out with R version 4.2.0.

## 3. Results

### 3.1. Literature Search and Summary of Published PopPK Models

The literature search identified a total of fourteen papers detailing the population pharmacokinetics of MTX in children [11,18,19,20,21,26,31,32,33,34,35,36,37]. Among them, six studies included ALL patients greater than 13 years or infants and were excluded for further analysis [18,31,32,33,34,35,37]. In addition, one PopPK study was conducted on pediatric patients with osteosarcoma and was also excluded [38]. Finally, six PopPK models of MTX in ALL children were included for the external evaluation [11,19,20,21,26,36]. The demographics, clinical characteristics, and doses for the patients of the included studies are summarized in Table 1. The mean age for all the studies was 5.0–7.5 years. Three of the models evaluated were based on the data from Chinese children [19,20,26], and the others were developed for Spanish [21], American [36], and Mexican [11] children. The largest model was developed with data from 311 pediatric patients proposed by Gao et al. [19], while the smallest model was developed with data from 36 patients proposed by Hui et al. [20].

The key information (e.g., model structure and covariate model) of the included PopPK models were provided in Table 2. The disposition of MTX was described by a two-compartment model in five studies and a three-compartment model in one study [19]. Typical estimates for MTX clearance in the included studies ranged from 3.52 [36] to 7.73 [20] L/h, with the lowest value being reported in the Jonsson et al. study with a median patient age of 5.0 years old. In studies based on a two-compartment model, the typical central volume of distribution varied across different subpopulations, ranging from 7.5 [11] to 24.1 [36] L, with the lowest value being reported in a Medellin-Garibay et al. study. All models estimated the IIV associated with MTX clearance, with values ranging from 8.2% [11] to 109.0% [26]. The highest IIV in MTX clearance was reported in a Jonsson et al. [36] study. The PopPK model developed by Hui et al. study implemented an inter-individual variability on MTX clearance with an estimated effect of 14.9% [20]. Applied covariates varied among the models, but all models included body size descriptors (e.g., weight and BSA) related to clearance and/or volume of distribution. Other identified covariates were eGFR and Scr on clearance, age on clearance and volume terms [21], gender, and pre-chemotherapy alkalinization volume on clearance [26]. As for residual unexplained variability, the applied forms included additive (including the additive error on the natural log-transformed concentrations), proportional, or combined proportional and additive error models with values ranging from 19.0% to 35.4% for the proportional component and 0.0035 to 0.0872 μmol/L for the additive component.

### 3.2. Characteristics of the Evaluation Dataset

**Figure 1:**
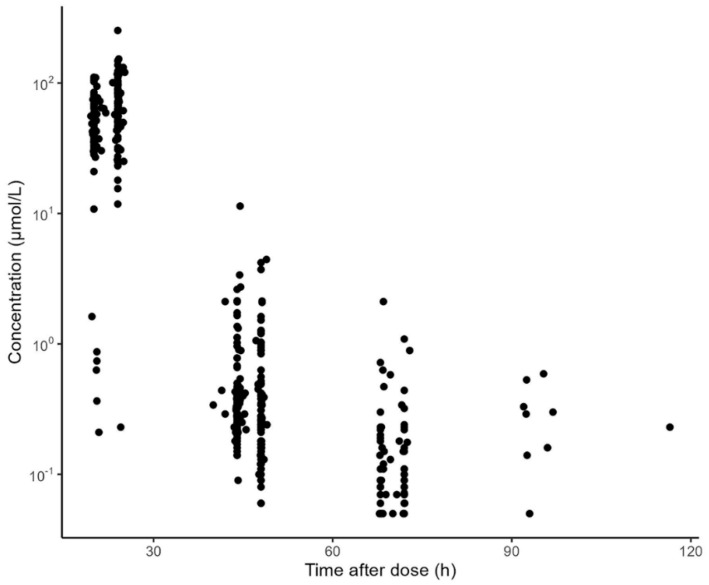
Methotrexate concentration versus time after dosing obtained with data available for external validation of the identified models.

The data used for external evaluation were collected from 51 ALL pediatrics with 354 concentration measurements. The demographics and clinical features of the included patients are summarized in Table 1. The prescribed HD-MTX dose varied from 1.0 g/m^2^ to 5.0 g/m^2^. The median age, weight, and BSA of the pediatric population were 5.0 years, 19.0 kg, and 0.77 m^2^, respectively. All the patients had normal renal function with a median eGFR of 149.9 mL/min/1.73 m^2^. The concentration observations versus time after dosing are displayed in Figure 1. In general, the measurements were mainly obtained at approximately 24, 48, 72, and 96 h after the start of MTX infusion.

### 3.3. Model Evaluation

**Figure 2:**
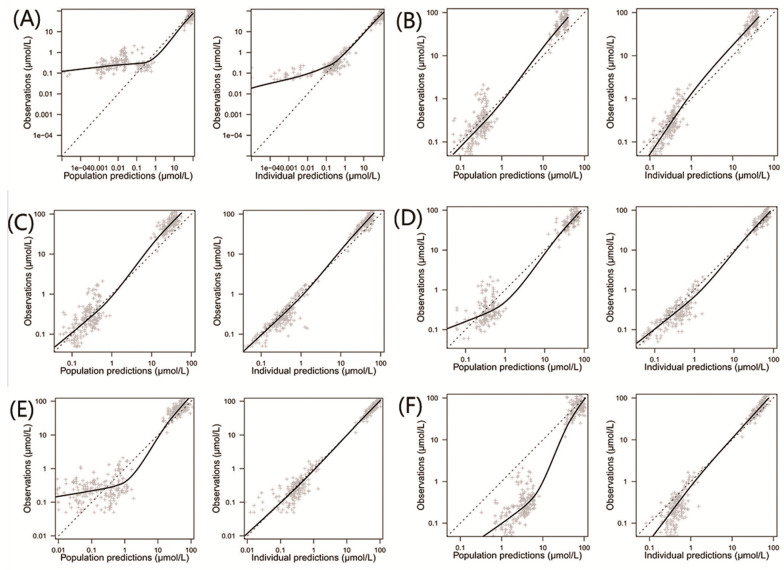
Observed concentrations versus population and individual predictions for the included population pharmacokinetic models of methotrexate in children with acute lymphoid leukemia. (**A**) Model proposed by Aumente et al. [21]; (**B**) model proposed by Gao et al. [19]; (**C**) model proposed by Hui et al. [20]; (**D**) model proposed by Medellin-Garibay et al. [11]; (**E**) model proposed by Zhang et al. [26]; and (**F**) model proposed by Jonsson et al. [36].

The comparisons of population and individual predictions to observations for each model are shown in Figure 2. For population predictions, two of the models (Aumente et al. and Zhang et al.) had many predictions underestimated compared to the observations (Panels A and E) [21,26], while the model proposed by Jonsson et al. showed significant overpredictions (Panel F) [36]. The systematic bias of the individual predictions for the above models almost disappeared, except for the model proposed by Aumente et al. (Panel A) [21], indicating a better accuracy of individual predictions compared to the population predictions. Additionally, no systematic bias was observed for the models proposed by Gao et al., Hui et al., and Medellin-Garibay et al. in terms of the population and individual predictions (Panels B, C, and D) [11,19,20].

**Figure 3:**
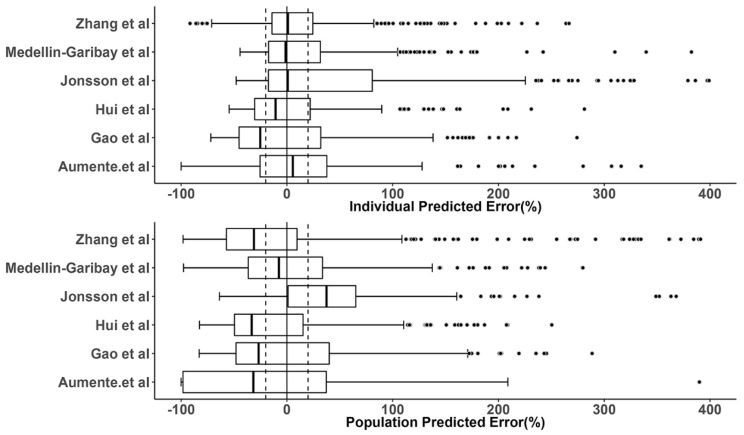
Prediction error (PE) distributions of the individual (upper panel) and population (bottom panel) predictions for evaluated population pharmacokinetic models of methotrexate using an independent dataset in children with acute lymphoid leukemia. In the boxplots, the lower boundary of the box indicates the 25th percentile, the line within the box marks the median, and the upper boundary of the box represents the 75th percentile. Whiskers above and below the box indicate 1.5-fold of the interquartile range below the 25th percentile and above the 75th percentile, respectively. Points above and below the whiskers are defined as outliers. The vertical black solid line represents the PE value at zero (unbiased), and the dashed black lines indicate ±20% of PE (acceptable bias) [11,19,20,21,26,36].

**Figure 4:**
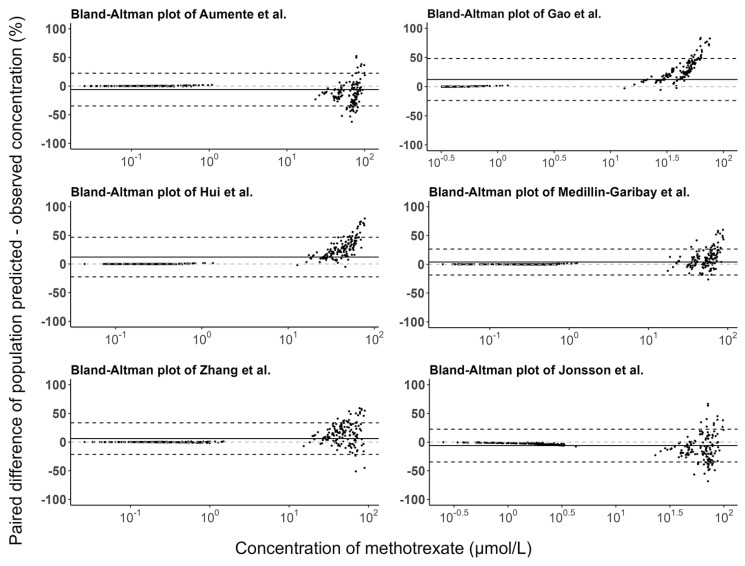
Bland-Altman plots of the prediction errors of population predictions for published population pharmacokinetic models of methotrexate based on an independent dataset in children with acute lymphoid leukemia. The black line is the mean of the population prediction error, and the black dashed lines are the mean ± 1.96 times the standard deviation of the population prediction error [11,19,20,21,26,36].

**Figure 5:**
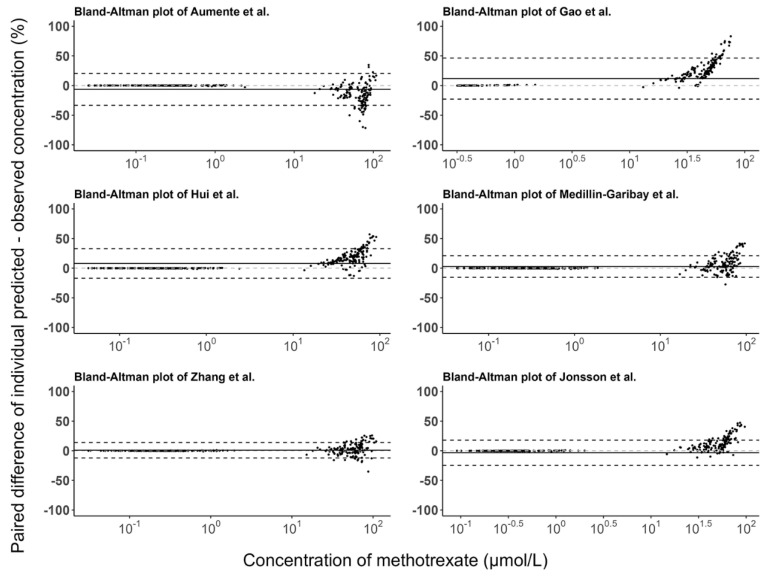
Bland-Altman plots of the prediction errors of individual predictions for published population pharmacokinetic models of methotrexate based on an independent dataset in children with acute lymphoid leukemia. The black line is the mean of individual prediction error, and the black dashed lines are the mean ± 1.96 times the standard deviation of the individual prediction error [11,19,20,21,26,36].

The prediction errors of the population and individual predictions for the evaluated models are visualized in box plots (Figure 3), and the median PE and MPE values are presented in Table 3. The median PEs of the population predictions for most of the models (except the Medellin-Garibay et al. study) were beyond ±20%, and the highest median PE (442.0%) was observed for the model proposed by Jonsson et al. [36]. Conversely, the median PE of the individual predictions for only one model (Gao et al. study) is distributed slightly over ±20% [19], and all other models showed acceptable bias. In terms of the MPE, the top three models showing good performances (<±20%) for both population and individual predictions were the Gao et al. [19], Hui et al. [20], and Zhang et al. [26] studies. Again, the model proposed by Jonsson et al. showed the worst bias with an MPE value of 780.87%. Bland-Altman plots showed that there was no apparent trend in the prediction errors of the population (Figure 4) and individual (Figure 5) predictions for four models (Aumente et al., Medellin-Garibay et al., Zhang et al., and Jonsson et al.) [11,21,26,36] over the whole concentration range observed in our data, while obvious trends were observed for the models proposed by Gao et al. [19] and Hui et al. [20]. All models show high RMSE with determined values > 30% (Table 3). The model proposed by Gao et al. performed best in RMSE in view of both the population (72.39%) and individual (63.96%) predictions [19]. This model also showed the smallest bias, as indicated by the MPEs. In general, the high RMSEs indicate the high imprecision of the models. That is, when validated with external data, the models all showed the need for further refinement.

## 4. Discussion

External evaluation is a crucial step to assess the accuracy, precision, and predictive performance of a PopPK model before its application in clinical practice. This step evaluates the model’s ability to describe the intended population and the impactful clinical variables related to the drug’s PK. This study is the first to review the published PopPK models of MTX in ALL pediatric patients and to systemically evaluate the predictive performance of these models using a new independent dataset.

All the evaluated models incorporated the influence of body size (e.g., body weight and BSA) on either clearance or volume of distribution. As MTX distributes into extracellular fluid and tissues, it is explainable that its volume of distribution tends to increase with the patient’s body size. As a predominantly renally excreted drug (80–90% of the administered dose), the descriptors of renal function (e.g., Scr and eGFR) are expected to influence MTX clearance. Of the evaluated models, only two of them included the influence of a renal function descriptor on MTX clearance [19,20]. This may be because most of the studies were based on patients with normal to moderately impaired renal function, and the impact of renal function on MTX clearance could not be detected significantly. The conflicting findings in the literature about the effect of renal function descriptors on MTX clearance might be arising from the following reasons: (1) the sample size of some studies is too small to detect the potential effect; (2) some studies only tested the effect of serum creatinine whereas eGFR is theoretically more predictive; and (3) the effect of renal function descriptors may be relatively weak in some studies and might have been blinded by other highly correlated covariates (e.g., age and body size-related covariates). Our patients in the validation dataset all had normal renal function, and the inclusion or the lack of inclusion of renal function in the PopPK model may be of little importance for the prediction.

The predictive performance of the models evaluated in our study varied considerably, and all the tested models only partially met the predefined criteria. In general, most of the models (except the Jonsson et al. model) demonstrated good accuracy (<±20% or a bit higher, in terms of median PE and MPE) for both the population and individual predictions. This highlighted that these models would be useful for a priori dose adjustment using only the population parameters and identified covariates. However, for all models tested, the RMSEs were between 62.88–1182.24% for population predictions and 63.96–152.25% for individual predictions. This demonstrated that the use of population PK parameters and patient covariates alone without feedback concentrations from individual subjects is inadequate to make precise predictions. Incorporation of feedback measurements into the PopPK model could improve the precision of Bayesian forecasting. The PopPK models of Aumente et al. [21], Medellin-Garibay et al. [11], and Zhang et al. [26] showed the best predictive performance across all the different tests, including the graphic and numerical predictive performance assessment. All these three models were originally validated using the dataset from the patient cohort very similar to the population with which the model was developed. Although the Gao et al. [19] and Hui et al. [20] models showed comparable performance to the above models in terms of numerical assessment, they had obvious prediction bias over the concentration range, as indicated by the Bland–Altman plots. Notably, the Gao et al. [19] model was developed based on the largest dataset (311 children with 4517 measurements), but it did not show a better predictive ability. In this model, a three-compartment disposition rather than a two-compartment disposition model was used, while the residual unexplained variability of the final model is still high (35.4%). We noted that the central volume of distribution in the Gao et al. [19] model (20.7 L) is higher than in other models (Table 2), leading to an obvious underprediction for the peak concentrations of MTX (Supplementary Table S1). Similarly, significant underprediction of peak MTX concentrations was also seen with the Hui et al. model (Table S1). The worst predictive performance was observed with Jonsson et al. model, especially for the population predictions. This is because a large variability (109%) was found for MTX clearance in this model, and probably the most influential covariates were not identified to reduce the variability. As expected, a better performance was found for the individual predictions after the incorporation of the IIVs of the population parameters. The model performance could be further improved when the trough concentrations were removed (Table S1), indicating that the model is inaccurate for predictions of trough concentrations. These results highlighted that it is essential to evaluate a model in target patient populations before its application in clinical practice.

We consider that the following factors can partially explain the heterogeneous performance of the included models. First, the incorporated covariates may affect the prediction performance. Theoretically, the models including the largest number of covariates could show better results in the external evaluation, especially for the population predictions. For instance, the Jonsson et al. model only considered the effect of body weight on a drug’s PK, which seems to be significantly insufficient for our data [36]. Furthermore, extrapolation of the covariate effects beyond the range of the values from the original patient population could lead to biased model predictions. Likewise, the lack of inclusion for important covariates in the PopPK models may result in high prediction errors for the population predictions (e.g., the Jonsson et al. model) [36]. Second, the differences in clinical characteristics of the external validation dataset and the patient populations in published PopPK models may influence the evaluation results. In this study, although we ensured that the mean age of the model evaluated was close to that of the validation dataset, the other patient characteristics were not considered. Differences in other parameters, such as disease severity, leucovorin rescue procedure, ethnicity and genetic polymorphisms, duration of MTX treatment, or co-medication, may alter the population parameters of the patient populations with similar age (e.g., the high central volume of distribution in Gao et al. study) [19], thus they may partly explain the models’ non-applicability to our patient cohort. Third, some of the constructed models were mainly based on peak and trough concentrations [19,20], and this would lead to a biased estimation of the distribution parameters. Likewise, our validation dataset is composed of peak and trough measurements. The concentrated distribution of such data points failed to fully assess the model prediction performance over the whole concentration-time range, potentially producing a biased evaluation.

The diverse landscape in the performance of the evaluated models observed in our study demonstrated that it is important to first execute extensive model evaluation before adopting a model in clinical facilities. Our study showed that the PopPK models of MTX failed to predict concentration-time data properly for external subjects that belong to a similar population in which the model was created. Therefore, it warns us that we should be cautious to apply the internally validated model to external institutions.

The present study has some limitations. First, the validation dataset was retrospectively collected from clinical settings, and uncertainty was associated with data records. Although we performed a data inspection by two independent persons, some random errors may still remain in the data. As the selected model is intended to be used in daily clinical practice, and the same random errors (e.g., recording errors) may also present in real situations, evaluation of the model performance using such real-world data is a meaningful and rigorous examination in order to test the robustness of the model. Second, the evaluation dataset was from a single center and consisted of centralized measurements (peak and trough), which may prevent a good appreciation of the drug’s distribution. Yet, the ultimate objective of the model evaluation was to use the selected models for MIPD in daily practice with only the peak and/or trough concentrations, thus it was crucial to test the models under this real-life condition.

## 5. Conclusions

The present study externally evaluated six published PopPK models of MTX in ALL children with independent clinical data. Some of the published PopPK models were found to have reasonably low levels of bias for both population and individual predictions but a relatively high degree of imprecision. The fact that none of the evaluated models passed the external validation emphasized that extensive validation is required before the adoption of the model used for clinical care for ALL pediatrics.

## Figures and Tables

**Table 1:** Demographics and clinical characteristics of the included studies and evaluation dataset of high-dose methotrexate in children with acute lymphoblastic leukemia (ALL).

Characteristics	Aumente et al. [21]	Gao et al. [19]	Hui et al. [20]	Medellin-Garibay et al. [11]	Zhang et al. [26]	Jonsson et al. [36]	Evaluation Dataset
Tumor Type	ALL	ALL	ALL	ALL	ALL	ALL	ALL
N	37	311	36	41	96	304	51
M/F	17/20	197/114	23/13	NA	66/30	NA	40/11
Age (years)	5.0 (0.5–17.0)	5.0 (0.75–15.2)	5.3 (1.3–15.8)	5.0 (1.0–15.0)	7.4 ± 3.1	5.0 (0.4–17.8)	5.5 (1.0–13.0)
Weight (kg)	24.2 (7.5–80.0)	19.0 (4.5–113.0)	18.4 (10.4–57.8)	21.2 (8.0–57.3)	26.1 ± 10.4	19.0 (5.8–93.3)	19.0 (9.5–62.0)
Height (cm)	115 (69–174)	112 (67–175)	107 (77–176)	115 ± 24	120 ± 21	110 (63–192)	113 (73–168)
BSA (m^2^)	1.07 (0.30–1.90)	NA	0.74 (0.47–1.64)	0.79 (0.41–1.6)	0.97 ± 0.29	0.76 (0.31–2.19)	0.77 (0.41–1.60)
MTX dose (g/m^2^)	1.23–5.23	1–5	2–5	2.89 ± 0.9	2–3.5	5–8	1–5
ALT (IU/L)	NA	16.0 (2.0–390.0)	16.5 (5.0–247.0)	19.1 (7.2–98.2)	32.7 ± 44.5	31.2 (6.0–228.6)	32.5 (4.0–250.0)
AST (IU/L)	NA	26.0 (8.0–135.0)	NA	25.8 (13.9–112.7)	45.3 ± 34.5	NA	36.0 (17.0–131.0)
Scr (mg/dL)	0.5 (0.3–0.8)	0.3 (0.1–1.5)	0.362 (0.11–1.07)	0.37 ± 0.11	0.36 ± 0.10	NA	0.31 (0.16–0.71)

The values are presented as median (range) or mean ± standard deviation. M/F: male/female; N: number of patients; ALL: acute lymphoid leukemia; BSA: body surface area; Scr: serum creatinine; MTX: methotrexate; ALT: alanine aminotransferase; AST: aspartate aminotransferase; NA: not available.

**Table 2:** Summary of the included population pharmacokinetics model of methotrexate for external evaluation.

Studies	Structural Model	Fixed Parameters and Covariate Models	IIV (%) (IOV (%))	RUV
Aumente et al. [21]	2 CMT	K_12_ (1/h) = 0.0155 K_21_ (1/h) = 0.0724 CL (L/h, age > 10 years) = 0.149 × Weight CL (L/h, age < 10 years) = 0.287 × Weight^0.876^ V_1_ (L, age > 10 years) = 0.437 × Weight V_1_ (L, age < 10 years) = 0.465 × Weight	K_12_ = 20.8 K_21_ = 35.2 CL = 41.7 V_1_ = 41.6	Prop = 16.2% Add = 0.0035 μmol/L
Gao et al. [19]	3 CMT	CL (L/h) = 6.9 × (Weight/19 kg)^0.75^ × (1 + (Scr-26) × (−0.0097)) × e^η^_CL_ V_1_ (L) = 20.7 × (Weight/19 kg) V_2_ (L) = 41.0 × (Weight/19 kg) × e^η^_V2_ Q_1_ (L/h) = 0.255 × (Weight/19 kg)^0.75^ V_3_ (L) = 3.17 × (Weight/19 kg) Q_2_ (L/h) = 0.217 × (Weight/19 kg)^0.75^	CL = 17.9 V_1_ = 26.2	Prop * = 35.4%
Hui et al. [20]	2 CMT	CL (L/h) = 7.73 × (BSA/0.735)^0.721^ × (eGFR × 1.73/192 × BSA)^0.256^ × e^η^_CL_^+IOV^ V_1_ (L) = 19.0 × (BSA/0.735)^0.985^ Q (L/h) = 0.283 × (Age/5.29)^0.278^ V_2_ (L) = 6.63 × e^η^_V2_	CL = 14.3 (14.9) V_2_ = 34.6	Prop = 30.2%
Medellin-Garibay et al. [11]	2 CMT	CL (L/h) = 6.5 × BSA^0.62^ × e^η^_CL_ V_1_ (L) = 0.36 × Weight × e^η^_V1_ Q (L/h) = 0.41 V_2_ (L) = 3.2 × e^η^_V2_	CL = 8.2 V_1_ = 25.9 V_2_ = 26.7	Prop = 20.1%
Zhang et al. [26]	2 CMT	CL (L/h) = (5.04 × (1 − 0.278 × Gender) × BSA^0.777^ + (OH/100)^0.514^) × e^η^_CL_ V_1_ (L) = 16.1 × e^η^_V1_ Q (L/h) = 0.203 × (Age/10)^1.56^ × e^η^_Q_ V_2_ (L) = 7.05 × (Age/10)^1.76^ × e^η^_V2_	CL = 49.6 V_1_ = 29.4 Q = 137.6 V_2_ = 107.7	Prop = 19.0% Add = 0.0872 μmol/L
Jonsson et al. [36]	2 CMT	CL (L/h) = Weight × 0.185 × e^η^_CL_ V_1_ (L) = Weight × 1.27 × e^η^_V1_ Q (L/h) = Weight × 0.017 × e^η^_Q_ V_2_ = Weight × 1.02 × e^η^_V2_	CL = 109.0 V_1_ = 26.0 Q = 22.0 V_2_ = 44.0	NR

* The exponential residual error model was used, in which the estimate is approximately equal to the proportional error model. CMT: number of compartments; K_12_: transfer rate constant from central compartment to peripheral compartment; K_21_: transfer rate constant from peripheral compartment to central compartment; CL: clearance; V_1_: central volume of distribution; V_2_ and V_3_: volume of distribution for peripheral compartments; Q: intercompartmental clearance; Scr: serum creatinine; eGFR: estimated glomerular filtration rate: BSA: body surface area; OH: pre-chemotherapy alkalinization volume; IIV: inter-individual variability; IOV: inter-occasion variability; RUV: residual unexplained variability; Prop: proportional residual unexplained error; Add: additive residual unexplained error; NR: not reported.

**Table 3:** Prediction error (PE) of the individual predictions (IPRED) and population predictions (PRED) to observations for the evaluated models.

Model	IPRED			PRED		
	Median PE (%)	MPE (%)	RMSE (%)	Median PE (%)	MPE (%)	RMSE (%)
Aumente et al. [21]	6.52	12.06	76.09	−31.70	−25.51	81.15
Gao et al. [19]	−25.20	1.33	63.96	−26.76	3.22	72.39
Hui et al. [20]	−10.43	8.78	82.96	−33.23	−8.24	62.88
Medellin-Garibay et al. [11]	−0.75	22.71	75.55	−7.56	6.39	68.33
Zhang et al. [26]	1.16	16.00	63.39	−29.92	15.62	145.92
Jonsson et al. [36]	5.25	64.44	152.25	442.04	780.87	1182.24

MPE: mean relative error, RMSE: root mean square error.

## Data Availability

All the relevant data are shown in the manuscript.

## Supplementary Materials

The following supporting information can be downloaded at: https://www.mdpi.com/article/10.3390/pharmaceutics15020569/s1, Table S1: Prediction error (PE) of the individual predictions (IPRED) and population predictions (PRED) to observations for the evaluated models for peak concentrations.
