# Supplementary material for: External Evaluation of Population Pharmacokinetic Models of Methotrexate for Model-Informed Precision Dosing in Pediatric Patients with Acute Lymphoid Leukemia
Source: Pharmaceutics. 2023 Feb 8;15(2):569. doi: 10.3390/pharmaceutics15020569 (PMC9962320; doi:10.3390/pharmaceutics15020569)
Supplement: Supplementary file 1 [file pharmaceutics-15-00569-s001.zip › pharmaceutics-2162979-supplementary.pdf]

Supplementary data

Table S1. Prediction error (PE) of the individual predictions (IPRED) and population predictions (PRED) to observations for the evaluated models for peak concentrations.

Model	IPRED			PRED		
	Median PE	MPE (%)	RMSE (%)	Median PE	MPE (%)	RMSE (%)
Aumente *et al*. [21]	19.27	32.47	53.62	25.56	34.90	58.31
Gao *et al*. [19]	-47.89	-43.23	47.02	-50.31	-44.48	48.44
Hui *et al*. [20]	-35.36	-30.59	35.64	-48.55	-45.28	49.27
Medellin-Garibay *et al*. [11]	-10.26	-6.70	28.12	-15.93	-9.56	32.72
Zhang *et al*. [26]	-4.30	-0.09	22.81	-22.42	-15.97	40.71
Jonsson *et al*. [36]	-20.39	-18.38	26.77	18.33	29.65	58.19

MPE: mean percentage error, RMSE: root mean square error.
